# Scutellarin protects cortical neurons against neonatal hypoxic‐ischemic encephalopathy injury via upregulation of vascular endothelial growth factor

**DOI:** 10.1002/ibra.12052

**Published:** 2022-07-09

**Authors:** Yu Zou, Chang‐Le Fang, Ya‐Ting Wang, Hua Li, Xi‐Liang Guo

**Affiliations:** ^1^ Department of Neurology The First Affiliated Hospital of Jinzhou Medical University Jinzhou China; ^2^ Liaoning Key Laboratory of Diabetic Cognitive and Perceptive Dysfunction Jinzhou Medical University Jinzhou China; ^3^ Animal Zoology Department Kunming Medical University Kunming China; ^4^ Hemodialysis Center Royal Adelaide Hospital Adelaide South Australia Australia

**Keywords:** hypoxic‐ischemic encephalopathy, neuroprotective effects, Scutellarin, siRNA, VEGF (vascular endothelial growth factor)

## Abstract

Neonatal hypoxic‐ischemic encephalopathy (NHIE) causes devastating cerebral damage and neurological deficits that seldom have effective therapies. This study aimed to explore the mechanisms underlying the therapeutic efficacy of Scutellarin in NHIE. NHIE models were successfully established. Zea‐longa score and triphenyte‐trazoliumchloride (TTC) staining demonstrated that hypoxia and ischemia (HI) insult induced prominent neurological dysfunctions and brain infarction. Protein microarray was applied to detect the differentially expressed genes in the cortex, hippocampus, and lung tissues of HI rats, which revealed the downregulation of vascular endothelial growth factor (VEGF) in these tissues. Additionally, double immunostaining uncovered VEGF expression was localized in the neurons. Besides, VEGF was decreasingly expressed in oxygen‐glucose deprivation (OGD) neurons, which was intriguingly reversed by Scutellarin treatment. Moreover, VEGF silencing increased OGD‐induced neuronal apoptosis and attenuated neurite outgrowth, which was enhanced by Scutellarin administration. GeneMANIA predicted a close correlation of VEGF with caspase 3, caspase 7, and interleukin (IL)‐1β, and qRT‐PCR revealed that Scutellarin treatment depressed the expression levels of them elevated in OGD neurons, but the Scutellarin‐depressed levels of these factors were prominently increased after VEGF silencing. Our findings suggested that Scutellarin exerted neuroprotective effects in NHIE potentially through mediating VEGF‐targeted inactivation of caspase 3, caspase 7, and IL‐1β.

## Graphical abstract

In this study, it was revealed that severe neurologic impairment and cerebral infarction were induced after hypoxia and ischemia insult, and microarray data unveiled differential expression of vascular endothelial growth factor (VEGF) in the cortex, hippocampus, and lung tissues, along with its colocalization with neurons. Though the expression of VEGF was decreased in oxygen‐glucose deprivation (OGD) neurons, it was obviously elevated after Scutellarin administration. Moreover, VEGF silencing depressed the effects of Scutellarin, which promoted neurite regrowth and attenuated cell apoptosis of OGD neurons. Thus, the efficacy of Scutellarin was partly abolished by interfering VEGF‐triggered upregulation of caspase 3,  caspase 7, and interleukin (IL)‐1β, which provided a new idea for the clinical therapeutics of hypoxic‐ischemic encephalopathy.

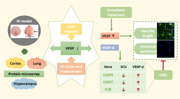

## 1 INTRODUCTION

Neonatal hypoxic‐ischemic encephalopathy (NHIE) is hypoxia‐induced perinatal brain injury manifested by cell damage, metabolic disorders, vascular regulation disorders, and cerebral flow reduction.^1^, ^2^ NHIE presents as the main cause of quick death among newborns and central nervous system sequelae, causing a great burden to the family and society.^3^ It was estimated that thousands of patients suffered the serious complications of NHIE, including learning dysfunction, epilepsy, cerebral palsy, and mental retardation.^4^, ^5^, ^6^ To date, current strategies for clinical therapies involve anticonvulsants, hypothermia, electrolyte, and fluid management, as well as atropine and adrenaline applications. Nevertheless, there is still a lack of effective therapeutic approaches, primarily as a result of lacking mechanistic understanding of the pathophysiology of NHIE.^7^, ^8^, ^9^ Therefore, the effective prevention and control measures in the study of the pathogenesis of NHIE should be urgently identified and developed.^10^, ^11^, ^12^

Scutellarin is a natural herbal medicine, which has been shown to treat inflammation, allergy, and bacterial and viral infections.^13^, ^14^, ^15^ Scutellarin has been successfully applied in the clinical treatment of neurodegenerative and ischemic diseases, including acute cerebral infarction and paralysis.^16^, ^17^ Previous work indicated that Scutellarin ameliorated the brain damage after NHIE, not only by attenuating brain edema but also by reducing HIE‐induced glutamate increases.^18^, ^19^ Therefore, Scutellarin exerted prospects as a neuroprotective medicinal herb against NHIE. Nevertheless, the molecular mechanism underlying its efficacy remains ambiguous.

Vascular endothelial growth factor (VEGF), recognized as a critical signaling protein involved in vasculogenesis and angiogenesis, has been verified to act as a crucial part in tumor growth and stimulating neo‐angiogenesis.^20^, ^21^, ^22^ In addition, VEGF can promote endothelial cell mitogenesis and cell migration, as well as enhance microvascular permeability.^23^, ^24^ Nowadays, VEGF has been reported with neuroprotective effects, and the expression level of VEGF after ischemic injury is significantly increased, indicating that VEGFs are key factors of tissue repair after HI injury.^25^, ^26^, ^27^ Either the important neuroprotective effect of Scutellarin in the NHIE recovery or the role of VEGF in the efficacy of Scutellarin in NHIE requires further exploration. In this study, we established in vivo and in vitro HI models to investigate the roles of crucially differentially expressed gene (DEG)—VEGF involved in the neuroprotective efficacy of Scutellarin exerting in HIE, providing novel insights into the treatment of NHIE.

## 2 MATERIALS AND METHODS

### 2.1 Animal care

The pregnant SD rats, obtained from the Animal Centre of Kunming Medical University, were kept in separate cages at a temperature (21–25°C) and humidity (45%–50%)—controlled room with 12 h light/dark alternating. Food and water were available to all animals. After birth, 7‐day‐old pups were used to establish the neonatal hypoxia and ischemia (HI) brain injury model. A sterile environment ought to be maintained during the operation. In addition, animals should be kept in a warm place to maintain body temperatures after HI modeling. All the experiments were carried out in accordance with the care and use of laboratory animals promulgated by the Ministry of Science and Technology of the People's Republic of China. This study was approved by the Animal Care and Use Committee of Kunming Medical University (kmmu2019045) and was in compliance with the National Institutes of Health Guide for the Care and Use of Laboratory Animals.

### 2.2 Establishment of neonatal HI model

The 7‐day‐old newborn rats (weighing 13–15 g) were anesthetized with isoflurane and then disinfected with alcohol. First, the right common carotid was exposed after a midline incision was made on the ventral skin of the neck. After that, the right common carotid was blocked with Monopolar Microsurgery Electro Coagulator and later the incision was sutured. After the surgery, the rats were returned to their cages with their mothers for 1 h, and then put into a chamber containing 92% nitrogen and 8% oxygen for 2 h. After the operation, they were moved back to their mothers to rest. Rats in the sham group underwent the same procedures except for ligation of the right carotid artery.

### 2.3 Zea‐longa neurologic score

The degree of neurological deficit was estimated by Zea‐longa neurologic score. The scoring criteria include 0–4 levels: 0—point indicates that rats behave normally with no neurological deficit symptoms found; 1—point means the left forelimb cannot be fully flexed and is accompanied by a mild neurological deficit; 2—points denote that rats are not capable of going straight and fall sideways; 3—points means rats stand toppling and falling to the left side, indicating severe neurological deficits; 4—points suggest rats cannot independently walk with consciousness deficit.

### 2.4 Tissue harvest

Once deep anesthesia was achieved with 3% isoflurane, rats were perfused with 0.9% normal saline followed by 4% paraformaldehyde (20 ml, pH 7.4, 0.01 mol/L PBS, pressure < 110 mmHg). Afterward, brains were removed and fixed with 4% paraformaldehyde for immunofluorescence staining. For microarray analysis, rats in the sham and HI groups were anesthetized and perfused with normal saline. Thereafter, the cortex, hippocampus, and lungs were removed and stored at −80°C.

### 2.5 Triphenyte‐trazoliumchloride (TTC) staining

Cerebral infarction was measured by TTC staining. Rats were anesthetized with isoflurane at 48 h after injury and then brains were quickly removed and transferred into the −20°C refrigerator for 20 min. After freezing, the brains were cut into five coronal slices (2 mm each), and then incubated with 2% TTC solution (Sigma) at 37°C for 5 min. After dyeing, fixation with 4% poly formaldehyde was followed for 24 h and section images were captured on the next day.

### 2.6 Protein microarrays

Protein Extraction Reagent (KangChen. Cat.# KC‐415, China) was applied to extract the total protein, and a BCA kit (KangChen, Cat.# KC‐430, China) was used to determine the concentration of extracted protein in three kinds of tissues. Additionally, the samples were placed into the protein array membranes at room temperature for 1–2 h and then put into a blocking buffer for 30 min. Following washes with washing buffer, the membrane was rinsed again after incubation with diluted biotin‐conjugated antibodies at room temperature for 2 h, followed by a reaction with HRP‐conjugated streptavidin (1:1000) at room temperature for 2 h. Membranes were washed and later detected with X‐ray film. Cytokine's relative expression levels were built via signal intensities comparison. Besides, densitometry was used to quantify the signal intensities. The consequences of distinct membrane comparisons were normalized by positive controls.

### 2.7 Immunofluorescence staining in vivo

Double immunofluorescence staining with NeuN and VEGF was performed in the cortex and hippocampus to explore the expression changes of VEGF in neurons after HI. Harvested tissues were dehydrated in a 30% sugar solution overnight and then cut into 15‐μm‐thick slices with a freezing microtome (Leica CM1900). Briefly, after incubating in 5% goat serum for 1 h at 37°C, sections were rinsed three times in PBS. Then, sections were incubated with primary antibodies of VEGF (Mouse; 1:50; ZSGB‐BIO; ZS‐5070), NeuN (Rabbit; 1:100; ZSGB‐BIO) for another 18 h at 4°C and then washed by PBS again. Subsequently, the following secondary antibodies (DyLight 488, Goat‐anti‐rabbit, 1:200; DyLight 594, Goat‐anti‐mouse, 1:200) were added for 2 h, which were then hatched with DAPI (Beyotime Biotechnology) for another 5 min. Five fields were obtained by Leica AF6000 DMI6000B (LAS AF system).

### 2.8 Primary cortical neuron cultures and PC12 cells

Cortical tissues were collected from 1‐day‐old neonatal pups. The harvested tissues were then cut up and digested with 0.25% trypsin at 37°C for 10 min. Subsequently, an equal amount of complete medium was taken to suspend digestion. The cells were collected after centrifugation at 1000 rpm for 10 min and then resuspended by 10% FBS. Afterward, 5 × 10^5^ cells/ml were coated on a 6‐well plate (Corning) containing 5% CO_2_. After culturing cells with 5% CO_2_ at 37°C for 4 h, the complete medium was substituted with neural basal cell medium (Invitrogen) containing 2% B27. Twenty‐four hours later, medium change was done for the first time, and then half change was refreshed every 3 days. PC12 cells were obtained from American Type Culture Collection and cultured in Dulbecco's modified Eagle medium (DMEM, Cat.# No. 10569‐010) supplemented with 10% fetal bovine serum (FBS, Cat.# No. 16000‐077) according to its recommended guidelines.

### 2.9 Construction of OGD model

After cortical neurons were cultured for 7 days, OGD was performed to mimic the HI condition in vitro. Briefly, the cell culture medium was replaced with a glucose‐free DMEM medium (Gibco), and neurons were placed in an anaerobic chamber with 5% CO_2_ and 95% N_2_ at 37°C for 1 h. Subsequently, cells were returned to the original medium, then placed in a normoxic chamber with 95% air and 5% CO_2_ for 16 h before further testing. Apart from the OGD exposure, the same procedures were carried out in the normal group.

### 2.10 Screening of effective siRNA fragments

First, VEGF genetic data were collected from National Center for Biotechnology Information (NCBI) at http://www.ncbi.nlm.nih.gov. Three specific small interfering RNAs (siRNAs) that inhibited the expression of the VEGF gene were produced and synthesized from Ribobio Company, along with one nonsense siRNA used as a negative control. PC12/neuron cells were separated into normal group, NC group, Reagent group, F1 group, F2 group, and F3 group at random. In brief, when PC12/neuronal cells grew to 40% confluence, they were transferred into a culture medium comprising siRNA fragments. The efficacy of siRNAs on VEGF mRNA expression was evaluated by qRT‐PCR, among which the most effective one was chosen for the following trials.

### 2.11 Transfection of siRNA

The transfection of siRNA was processed according to the operation manual. Then the reaction system was set as follows according to instructions: 60 µl 1Xbuffer + 5 µl siRNA +5 µl reagent was added to one tube, stewing for 15 min at room temperature, which was then added to the cell culture medium. After transfection for 72 h, the successful transfection of siRNA was observed via red Cy3 fluorescence labeling.

### 2.12 Drug administration

Cells were assigned to four groups: normal group, OGD group, OGD + SCU + NC group, and OGD + SCU + VEGF‐si group. First, 150 mg Scutellarin provided by Longjin Pharmaceutical Co., Ltd was dissolved in 1 ml DMSO. The cells were pretreated with 3 μM Scutellarin for 24 h and then subjected to OGD for 1 h, followed by immediate reoxygenation in the neurobasal medium with 3 μM Scutellarin addition for 24 h at 37°C. Nothing was added in the case of the normal group and 3 μM 1/3000 DMSO was administered to the vehicle‐treated groups in the same manner.

### 2.13 QRT‐PCR

Total RNA was extracted using Trizol reagents and then transcribed reversely to cDNA. Then the reaction was performed in a mixtures system composed of 0.6 µl upstream primer, 0.6 µl downstream primer, 1 µl cDNA, 7.8 µl PCR nuclease‐free water, and 10 µl 2 × PCR master mix. The primer sequences of PCR products are shown in Table 1. Glyceraldehyde phosphate dehydrogenase (GAPDH) was used as an internal reference gene to normalize mRNA content for each sample. The qRT‐PCR reaction conditions were set as follows: denaturing at 95°C for 3 min, then 40 cycles of amplifying at 95°C for 30 s, annealing at 50°C for 30 s, and extension at 60°C for 30 s, along with a terminal elongation step at 60°C for 10 min. Relative expression for each sample was normalized with GAPDH values by the 2^−△△*Ct*^ method.

**Table 1:** Primer sequences of the detected genes

Genes	Forward sequences	Reverse sequences
Vascular endothelial growth factor	5′‐CCTTGCTGCTCTACCTCCAC‐3′	5′‐ACAAATGCTTTCTCCGCTCT 3′
Casp3	5′‐TTCTTCAGAGGCGACTACT‐3′	5′‐TCCCACTGTCTGTCTCAAT‐3′
Casp7	5′‐TCTTTGCTTACTCCACGGTT‐3′	5′‐ACCCTGGTCAGGATCTGCAT‐3′
Interleukin‐1β	5′‐GAGCTGAAAGCTCTCCACCT‐3′	5′‐TTCCATCTTCTTCTTTGGGT‐3′
GAPDH	5′‐CCTCAAGATTGTCAGCAAT‐3′	5′‐CCATCCACAGTCTTCTGAGT

Abbreviation: GAPDH, glyceraldehyde phosphate dehydrogenase.

### 2.14 Immunofluorescence staining in vitro

The prepared cell slices were washed in PBS four times and then incubated with 5% goat serum at room temperature for 1 h to block nonspecific binding. Afterward, cell slices were incubated with primary antibodies of Tuj1 (mouse; 1:100; Abclonal) overnight at 4°C. Then, after three washes with PBS, incubation with secondary antibodies (DyLight 488, anti‐mouse IgG; 1:200; ZSGB‐BIO) was followed for 1 h at 37°C. The images were acquired at 200 magnification under a fluorescent microscope (Leica, Germany). Five random fields were selected for quantification. Image‐Pro Plus 6.0 software (MediaCybernetics) was used to quantify the number of cells and the average length of neurites (50 neurons per cover‐slip, six independent experiments).

### 2.15 TUNEL staining assay

Neuron apoptosis after the OGD exposure was detected by in situ cell death detection kit, TMR red (Roche, Indianapolis, IN). After being fixed in paraformaldehyde for 10 min at 4°C, the slices were washed three times with PBS and then incubated in permeabilization solution for 8 min. Following being put in a TUNEL reaction mixture (In situ Cell Death Detection Kit; Roche Molecular Biochemicals, Mannheim, Germany) at 37°C for 1 h and moved to 0.1 mol/L PBS, followed by DAPI counterstaining of nuclei. Five random fields were selected for quantification. Photographs were visualized at 200 magnification under fluorescence microscopy (Leica), and the percentage of TUNEL/DAPI was measured using Image‐Pro Plus 6.0 software (50 neurons per coverslip, six independent experiments).

### 2.16 Statistical analysis

SPSS software 19.0 (IBM) was applied for statistical analysis. If the data meet the normal distribution, one‐way analysis of variance (ANOVA) shall be used with Bonferroni analysis for data under equal variance conditions or Dunnett's analysis for data under uneven variance conditions. All data were presented as mean ± standard deviation (SD). Kruskal–Wallis test was used for data that do not meet normal distribution. *p* < 0.05 value was considered statistically significant.

## 3 RESULTS

### 3.1 NHIE‐induced cerebral infarction and cerebral edema

**Figure 1:**
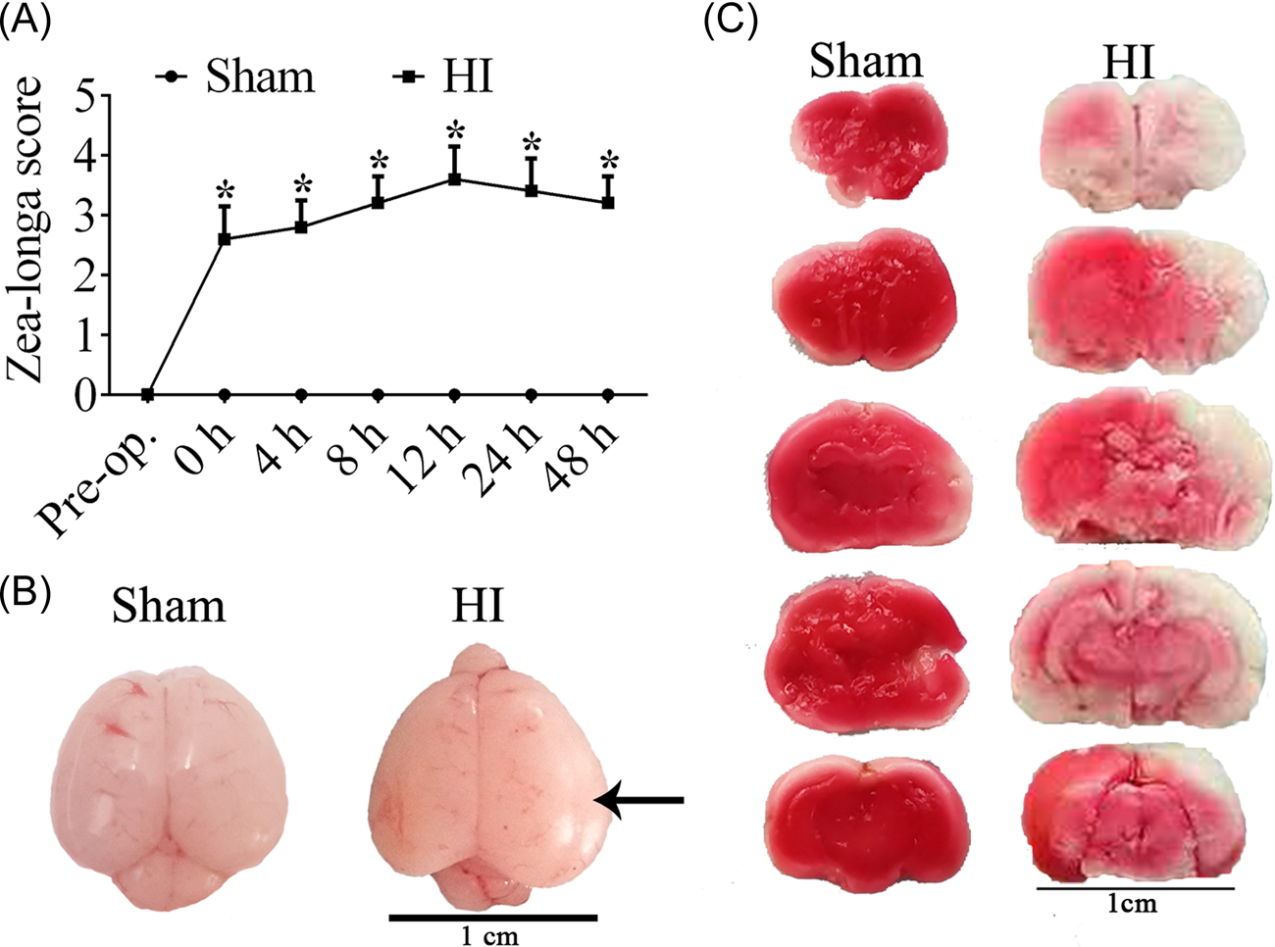
Symptoms of neonatal rats after HI. (A) The Zea‐longa score between HI and Sham group within 48 h. (B) The macroscopic cerebral images of neonatal rats after HI. (C) The TTC staining images of rats in HI and Sham groups. h, hours; HI, hypoxia‐ischemia; TTC, triphenyl tetrazolium chloride staining. Scale bar = 1 cm. The data were presented as mean ± SD. *p < 0.05, *n* = 5/group. [Color figure can be viewed at wileyonlinelibrary.com]

Zea‐longa score demonstrated that rats in the HI group had higher scores than those in the sham group within 48 h after HI insult, indicating severe neurological deficits induced in HI rats (Figure 1A, *p* < 0.05). Besides, HI‐induced brain edema was obviously observed by macroscopic images (Figure 1B). Meanwhile, the infarcted brain tissues were analyzed with TTC staining, of which the results exhibited a large pale area in the brain tissues of HI rats (Figure 1C). These proved HI‐induced neurological dysfunctions and cerebral damage, indicating the successful establishment of HI models in this study.

### 3.2 Detection of DEGs in the cortex, hippocampus, and lung tissues of HI rats

**Figure 2:**
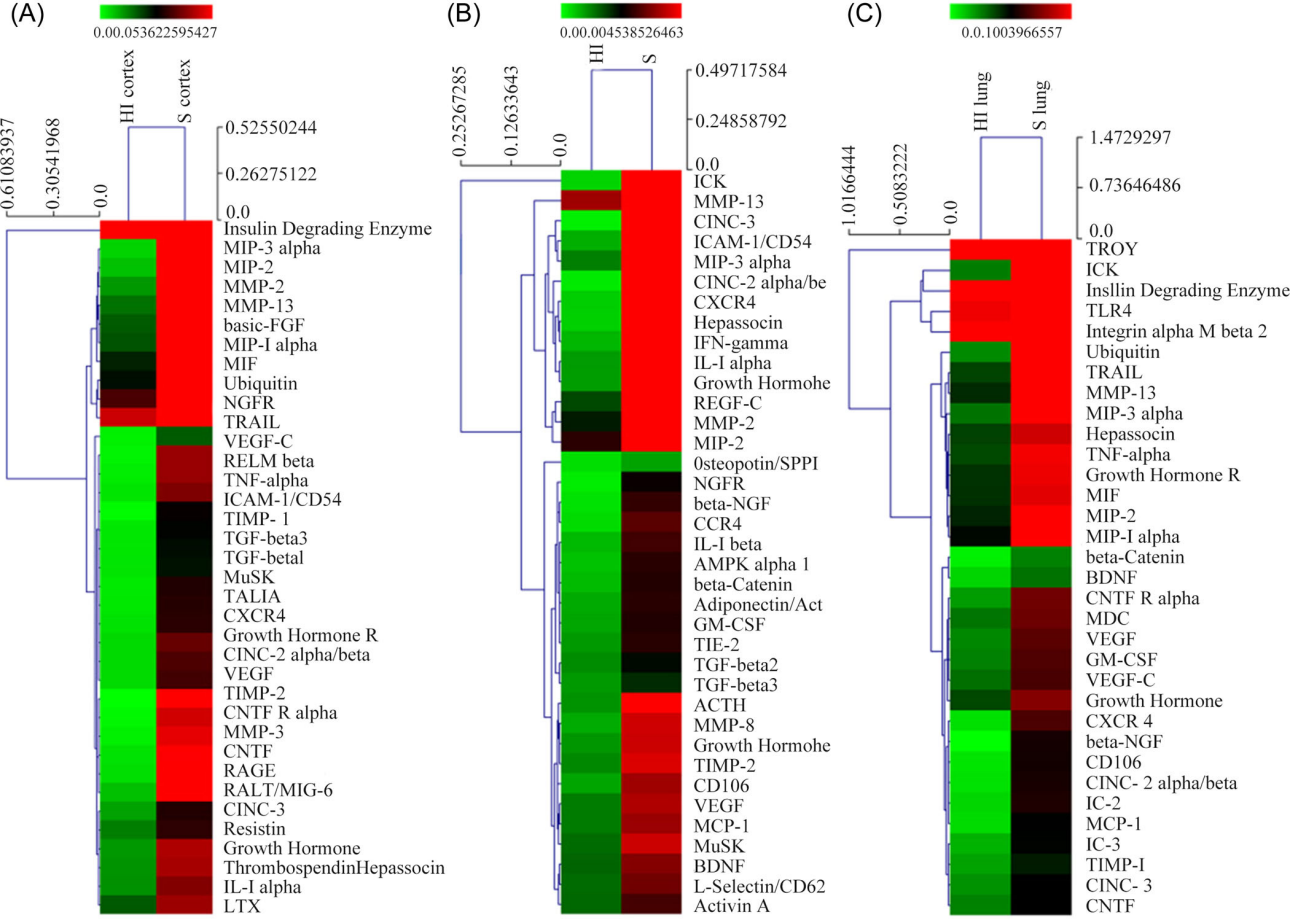
Detection of DEGs after HI. The heat map of DEGs in the (A) cortex, (B) hippocampus, and (C) lung tissues. Upregulated genes were labeled in red and downregulated genes in green. HI, hypoxia ischemia; DEGs, differentially expressed genes. [Color figure can be viewed at wileyonlinelibrary.com]

To elucidate the molecular mechanism underlying the pathogenesis of NHIE, we applied protein microarrays to primarily detect the expression changes of DEGs in the cortex, hippocampus, and lung of rats between HI and sham groups. As revealed, VEGF was found differentially expressed in the cortex, hippocampus, and lung tissues, and its levels were all downregulated in these tissues of HI rats (Figure 2A–C).

### 3.3 The expression and localization of VEGF in neurons

**Figure 3:**
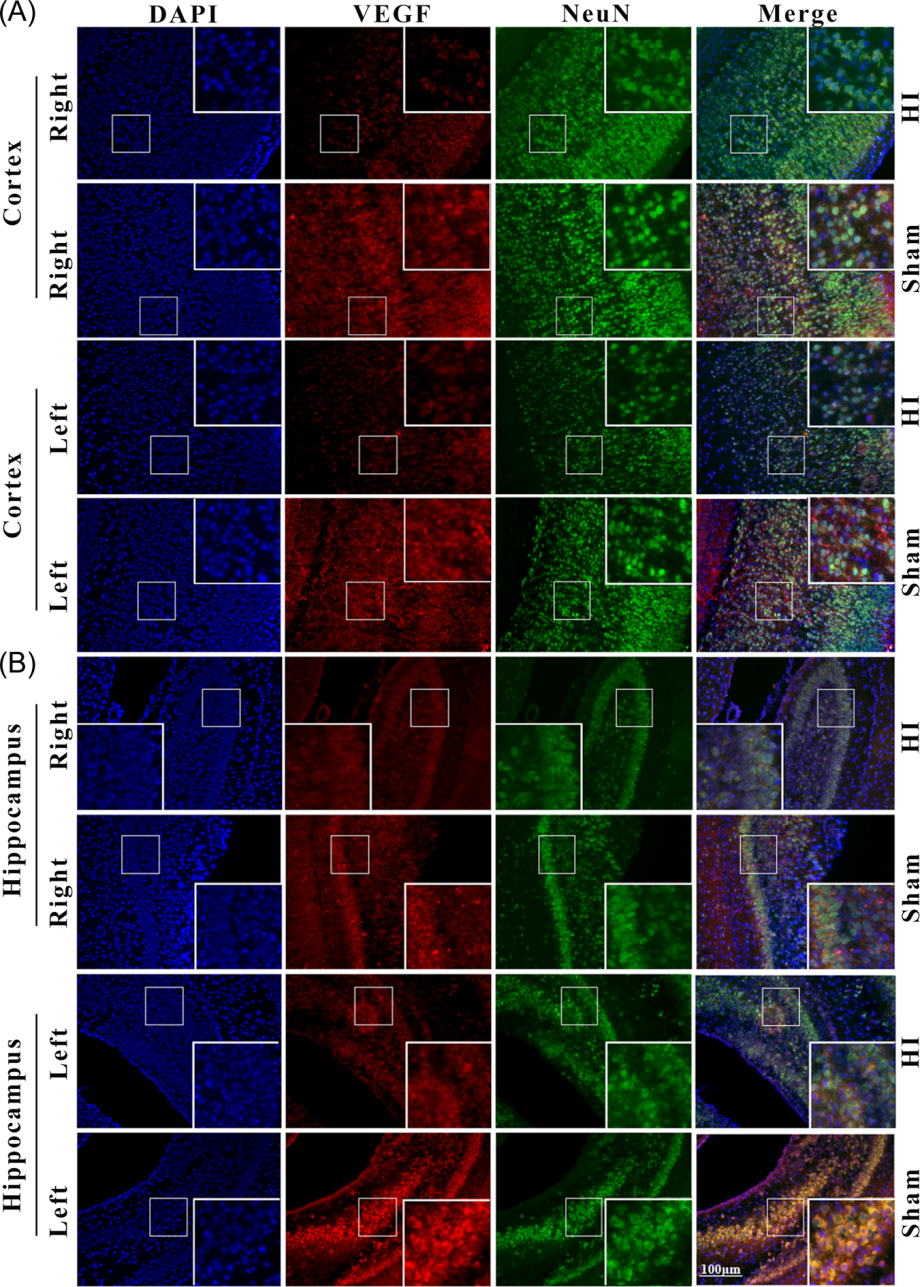
Double immunofluorescence staining of VEGF and NeuN in the cortex and hippocampus. (A, B) The expression and distribution of VEGF in neurons in distinctive tissues (right/left cortex, right/left hippocampus) in groups of HI and sham. The neuron was marked with NeuN (green), VEGF was in red, and the nucleus was marked with DAPI (blue). DAPI, 4′, 6‐diamidino‐2‐phenylindole; HI, hypoxia ischemia; NeuN, the neuronal nuclei specific marker; VEGF, vascular endothelial growth factor. Scale bar = 100 μm. [Color figure can be viewed at wileyonlinelibrary.com]

To evaluate the expression and localization of VEGF in the neurons of HI rats, immunofluorescence double staining with VEGF and NeuN was performed in the cortex and hippocampus tissues between sham and HI groups. It was observed that VEGF was abundantly expressed in the right cortex and hippocampus neurons in sham groups, which was obviously decreased after HI (Figure 3A,B). Moreover, the expression of VEGF was colocalized with NeuN in the left cortex and hippocampus (Figure 3A,B).

### 3.4 Effective siRNA fragment was successfully screened and validated

**Figure 4:**
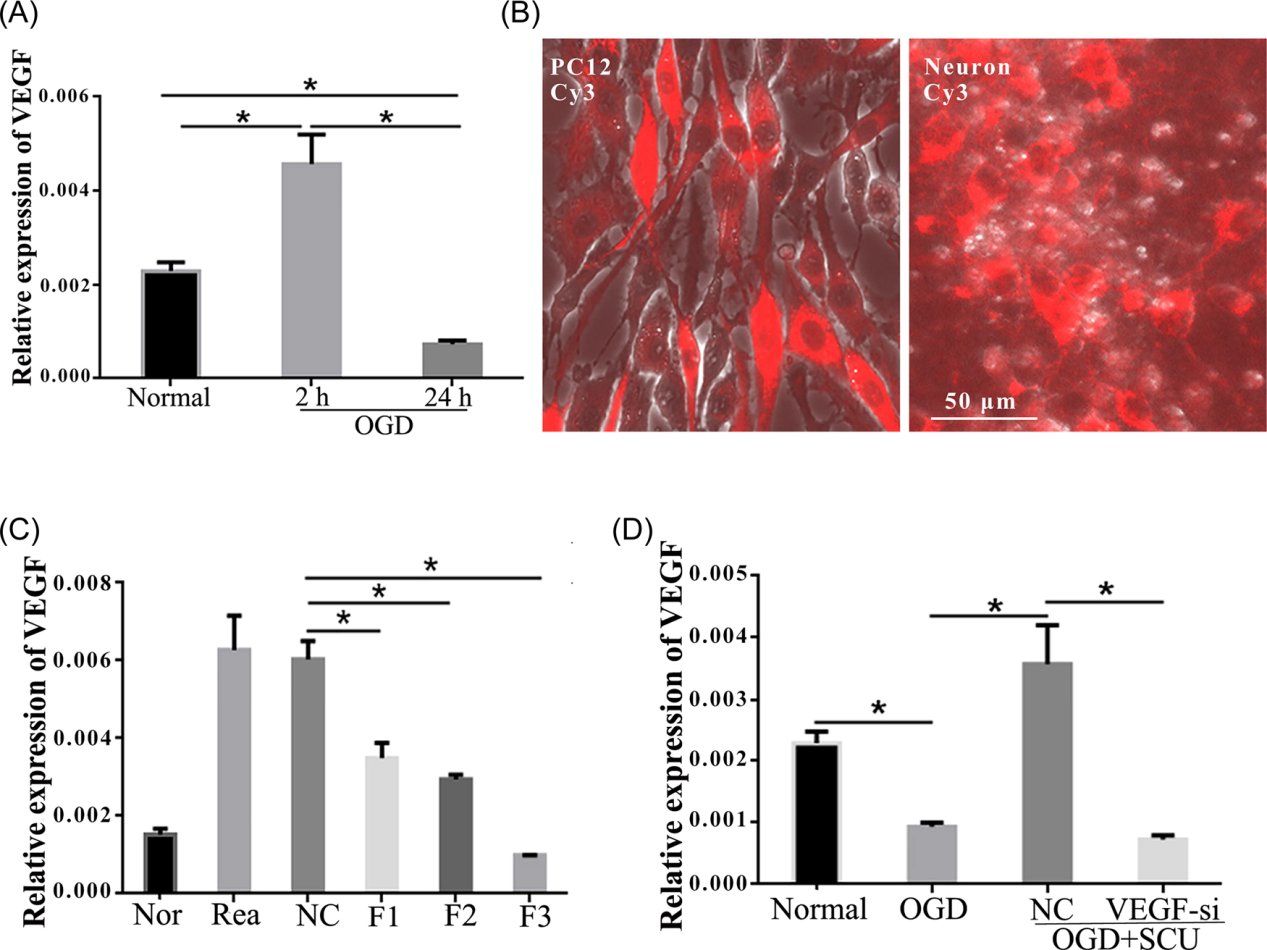
Expression of VEGF in OGD neurons with SCU administration and screening of VEGF interference fragments. (A) Quantitative histograms of the relative expression of VEGF in the groups of normal, OGD‐2 h, OGD‐24 h, and OGD + SCU. (B) Transfection of siRNAs into the PC12 cells and neurons. Then the siRNA production carrying the red Cy3 was observed by fluorescence microscope. (C) The expression levels of VEGF in each group to determine the effective siRNA fragment. (D) The levels of VEGF in normal, OGD, OGD + SCU, and OGD + SCU + VEGF‐si groups. F1, treatment with No.1 siRNA fragment; F2, treatment with No.2 siRNA fragment; F3, treatment with No.3 siRNA fragment; h, hours; HI, hypoxia‐ischemia; NC, negative control group; OGD, oxygen‐glucose deprivation; OGD‐24 h, 24 h after OGD; VEGF, vascular endothelial growth factor; SCU, scutellarin; VEGF‐si, silencing of VEGF. Scale bar = 50 μm. Data were presented as mean ± SD. *p < 0.05, *n* = 6/group. [Color figure can be viewed at wileyonlinelibrary.com]

We evaluated the expression changes of VEGF in the OGD neurons in vitro. The results indicated VEGF mRNA expression at 2 h after OGD was significantly higher than the normal group but prominently decreased at 24 h (Figure 4A, *p* < 0.05), which indicates that the VEGF was in a low expression status in the long term. F1‐F3 silencing fragments were used to depress VEGF production and were transfected into PC12 and neurons (Figure 4B). The results of qRT‐PCR showed three siRNA sequences; all could decrease the expression of VEGF, among which F3 exhibited the most effective silencing fragment (Figure 4C, *p* < 0.05).

### 3.5 Scutellarin facilitates neurite outgrowth in cortical neurons after OGD injury via VEGF

**Figure 5:**
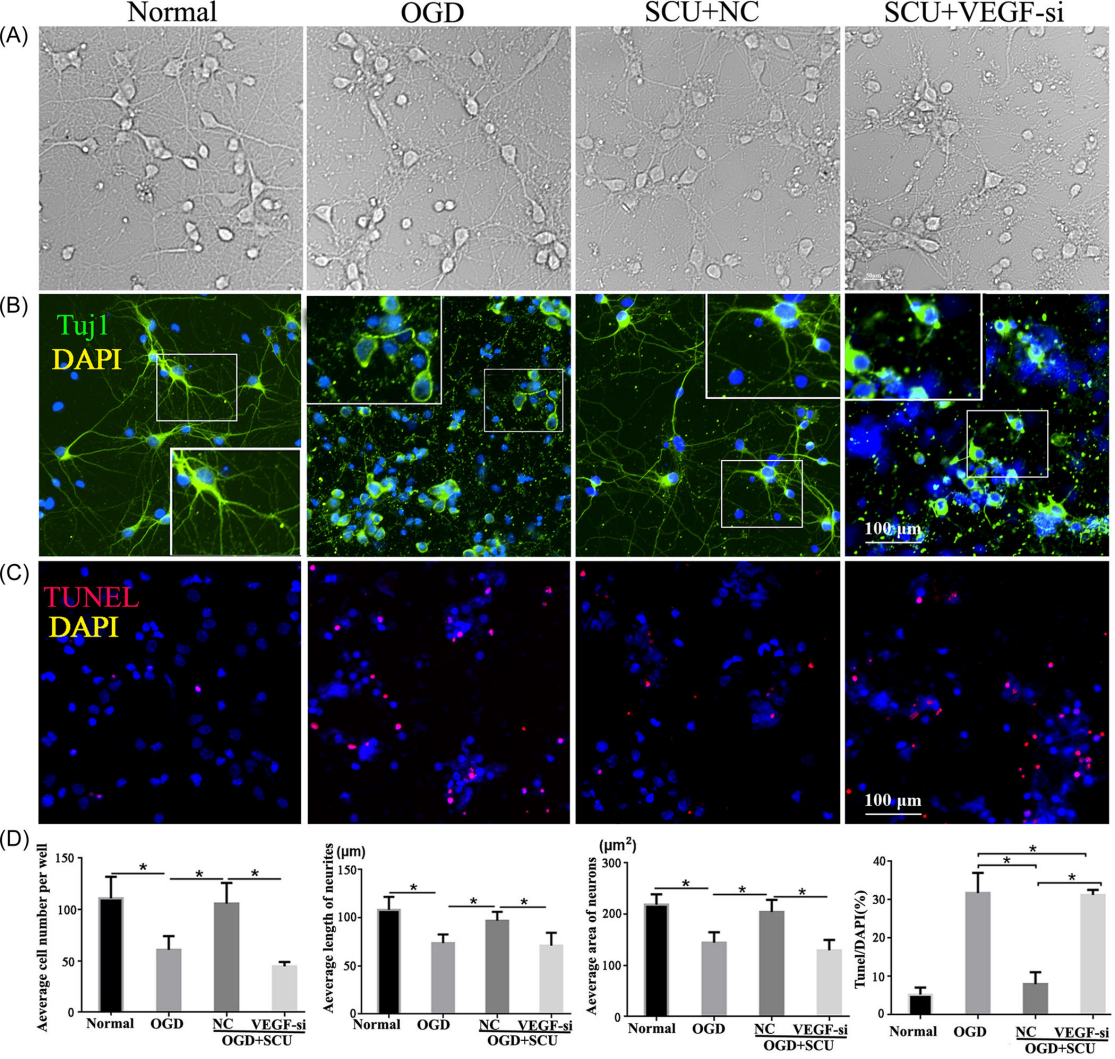
Effect of Scutellarin in the neuronal growth involving VEGF expression. (A) Neuronal morphology under the light microscope in the normal, OGD, SCU + NC, and SCU + VEGF‐si groups. (B) Immunofluorescence staining of Tuj1 and (C) TUNEL among the four groups. Tuj1 positive cells were shown in green, DAPI staining for the nucleus (blue), and TUNEL staining for apoptotic cells (red). (D) The average cell number, average area of neurons, average length of neurites, and apoptosis rate of cells in each group. DAPI, 4′, 6‐diamidino‐2‐phenylindole; NC, negative control group; OGD, oxygen‐glucose deprivation; SCU, Scutellarin; TUNEL, terminal deoxynucleotidyl transferase‐mediated dUTP‐biotin nick‐end labeling assay; VEGF, vascular endothelial growth factor; VEGF‐si, silencing of VEGF. Scale bar = 50 μm. Data were presented as mean ± SD. *p < 0.05, *n* = 6/group. [Color figure can be viewed at wileyonlinelibrary.com]

The expression levels of VEGF in OGD neurons were suppressed; however, when treated with Scutellarin, its levels were significantly elevated (Figure 4D, *p* < 0.05). Whereas the levels of VEGF in the SCU + VEGF‐si group were obviously lower than that in SCU + NC group (Figure 4D, *p* < 0.05). The bright‐field images of cortical neurons observed neurite damage and partial disappearance after OGD. After Scutellarin administration, neurites of neurons expanded, creating a broad network that was devastated by silencing VEGF (Figure 5A). Moreover, immunofluorescence staining of Tuj1 demonstrated that the number of neurons decreased, and neurons were more disorganized in the OGD group compared to the normal group. This situation was improved after Scutellarin administration (Figure 5B,D, *p* < 0.05). However, the number of neurons reduced with shorter neurites again after VEGF interfering (Figure 5B,D, *p* < 0.05). In addition, TUNEL staining demonstrated that the neurons were significantly augmented after Scutellarin administration and exhibited a significant reduction after the interference of VEGF (Figure 5C,D, *p* < 0.05). What's more, the quantitative data further validated that cell number, length of the neurite, and the area of neurons in the OGD + SCU + VEGF‐si group were less than those in the OGD + SCU + NC group. However, the apoptosis rate after VEGF interference was higher than the OGD + SCU + NC group (Figure 5D, *p* < 0.05).

### 3.6 VEGF silencing upregulated the expression levels of interleukin (IL)‐1β, caspase 3, and caspase 7 in SCU‐treated OGD neurons

**Figure 6:**
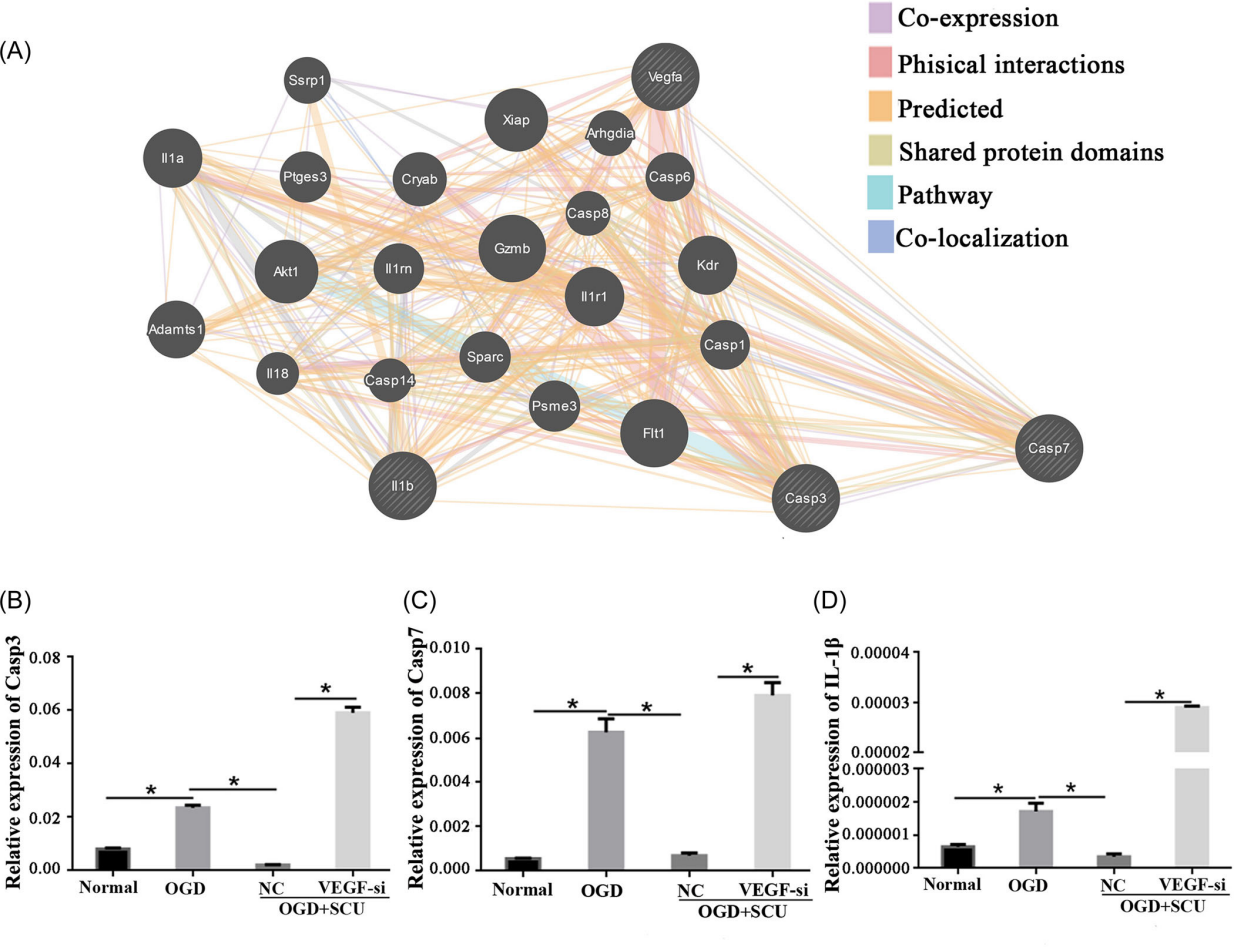
mRNA levels change factors interacting with VEGF. (A) Gene network diagram of VEGF, interleukin (IL)‐1β, caspase 3, and caspase 7 by GeneMANIA analysis. The purple line represents co‐expression relation; the blue line represents pathway relation. Quantitative histograms of the relative expression of (B) caspase 3, (C) caspase 7, and (D) IL‐1β. NC, negative control group; OGD, oxygen glucose deprivation; SCU, Scutellarin; VEGF, vascular endothelial growth factor; VEGF‐si, silencing of VEGF. Data were presented as mean ± SD. *p < 0.05, *n* = 6/group. [Color figure can be viewed at wileyonlinelibrary.com]

The interactive relationship of VEGF with other factors was analyzed on GeneMANIA. The results indicated a pathway and coexpression relationship among VEGF, IL‐1β, caspase 3, and caspase 7 (Figure 6A). The transcription of the latter three genes is involved in cell apoptosis. Next, qRT‐PCR data demonstrated that the expression of IL‐1β, caspase 3, and caspase 7 was significantly increased after OGD insult in comparison with that of the normal group, which was inhibited with Scutellarin treatment (Figure 6B–D, *p* < 0.01). Nevertheless, VEGF silencing elevated Scutellarin‐depressed levels of IL‐1β, caspase 3, and caspase 7 in OGD neurons (Figure 6B–D, *p* < 0.01). These outcomes suggested that Scutellarin might play a critical role in neuronal repair by mediating VEGF‐targeted, inactivation of IL‐1β, caspase 3, and caspase 7.

## 4 DISCUSSION

In this study, it was revealed that severe neurologic impairment and cerebral infarction were induced after HI insult, and microarray data unveiled differential expression of VEGF in the cortex, hippocampus, and lung tissues, along with its colocalization with neurons. Though the expression of VEGF was decreased in OGD neurons, it was obviously elevated after Scutellarin administration. Moreover, VEGF silencing depressed the effects of Scutellarin, which promoted neurite regrowth and attenuated cell apoptosis of OGD neurons. Thus, the efficacy of Scutellarin was partly abolished by interfering with VEGF‐triggered upregulation of caspase 3, caspase 7, and IL‐1β, which provided a new idea for the clinical therapeutics of HIE.

### 4.1 VEGF silencing inhibited axon growth and promoted cell apoptosis in OGD neurons with Scutellarin administration

In our study, it is revealed that the VEGF expression was decreased after HI by protein microarrays and qRT‐PCR verification. Besides, immunofluorescence staining of NeuN indicated that VEGF was localized in neurons. It has been suggested that VEGF may participate in hippocampal reorganization by promoting the growth of neurons.^28^ Moreover, studies have shown that VEGF overexpression played a neuroprotective role in promoting neurological recovery after HI in neonatal rats.^27^, ^29^ Our results consistently showed that Scutellarin promoted neuronal growth and reduced apoptosis. It has been demonstrated that Scutellarin has a protective effect on neuronal injury therapeutically.^30^ What's more, it also indicated that Scutellarin downregulated the ratio of apoptotic cells and restrained the generation of reactive oxygen species (ROS) in OGD‐induced primary cortical neurons in vitro.^31^ In our research, we found VEGF mRNA level was elevated in Scutellarin‐treated OGD neurons. Additionally, other research suggested that the VEGF protein expression was upregulated via adenovirus vector‐mediated HIF‐1 Alpha or VEGF gene therapy, which exerted neuroprotective effects by decreasing neuron apoptosis, increasing angiopoiesis, and improving the long‐term behavioral function.^32^, ^33^ In the present study, we found that interfering VEGF could attenuate neurites regrowth and increase cell apoptosis in OGD neurons with Scutellarin treatment. Thus, our results indicated that Scutellarin acts as a neuroprotective agent against NHIE involved in the expression of VEGF.

### 4.2 VEGF silencing promotes cell apoptosis via regulating caspase 3, caspase 7, and IL‐1β in Scutellarin‐treated OGD neurons

After interfering with VEGF in OGD neurons treated with Scutellarin, we found caspase 3, caspase 7, and IL‐1β were upregulated. According to the protein network, it was indicated that there is a regulatory relationship between VEGF and these inflammatory and apoptosis molecules. As is known, caspase 3 and caspase 7, members of the caspase (cysteine aspartate protease) family of proteins, have been considered executive proteins of apoptosis.^34^, ^35^ Some studies found that the levels of caspase 3 and caspase 7 were aberrantly activated in both cortices and hippocampi following HI.^36^, ^37^ In addition, IL‐1β, as one of the IL‐1 family of cytokines members, draws a significant impact on inflammatory responses and is involved in cell proliferation, differentiation, and apoptosis.^38^ What's more, some other research reported that Scutellarin significantly attenuated the expression of inflammatory, apoptosis factors.^39^ In our study, we also reported that Scutellarin is able to downregulate the expression of caspase 3, caspase 7, and IL‐1β. However, after interfering with VEGF, the expression of these factors was reversed again, indicating that VEGF plays a crucial role in the process of Scutellarin treatment associated with caspase 3, caspase 7, and IL‐1β. Overall, the antiapoptosis effect of Scutellarin may be attributed to caspase, IL‐1β inhibition, and VEGF activation.

## 5 CONCLUSIONS

This study identified the critical roles of VEGF, silencing of which depressed the protective effects of Scutellarin, decreasing the neuron growth and increasing the neuronal apoptosis. The underlying mechanisms might be potentially related to VEGF‐triggered upregulation of caspase 3, caspase 7, and IL‐1β.

## AUTHOR CONTRIBUTIONS

Xi‐Liang Guo and Hua Li designed the study; Yu Zou, Chang‐Le Fang, and Ya‐Ting Wang performed the experiments and statistical analysis and wrote the manuscript. All contributing authors approved the final version of the manuscript.

## CONFLICT OF INTEREST

Professor Hua Li is a member of Ibrain Journal editorial board and is not involved in the peer review process of this article.

## ETHICS STATEMENT

All the experiments were approved by the Animal Care and Use Committee of Kunming Medical University (kmmu2019‐045).

## Data Availability

The data used to support the findings of this study are available from the corresponding author upon request.
